# Transcriptomic identification of potential antioxidative enzyme regulators of the gametophytic-to-embryogenic switch in barley microspores

**DOI:** 10.3389/fpls.2025.1735720

**Published:** 2026-01-21

**Authors:** Anna Nowicka, Zbyněk Milec, Monika Krzewska, Przemysław Kopeć, Agnieszka Springer, Ewa Dubas, Iwona Żur

**Affiliations:** 1 The Franciszek Górski Institute of Plant Physiology Polish Academy of Sciences, Kraków, Poland; 2 Centre of Plant Structural and Functional Genomics, Institute of Experimental Botany (IEB), Czech Academy of Sciences (CAS), Olomouc, Czechia; 3 Laboratory of Ecological Plant Physiology, Global Change Research Institute, Czech Academy of Sciences (CAS), Brno, Czechia

**Keywords:** antioxidant defense, *Hordeum vulgare*, microspore embryogenesis, redox homeostasis, RNA-seq

## Abstract

Microspore embryogenesis (ME) relies on the cellular reprogramming of the default gametophytic developmental pathway, which normally directs microspores toward pollen formation, into an embryogenic pathway that leads to the development of embryo–like structures (ELS) and, subsequently, haploid or doubled haploid (DH) plants. To test how redox control underpins this switch, we have carried out an extended *in silico* analysis of previously published RNA-seq data from two barley cultivars differing in ME competence (Igri, responsive; Golden Promise, recalcitrant) across four early induction stages (0–III). A curated set of 472 antioxidant/redox genes—core detoxification enzymes, the ASC–GSH cycle, *TRX/GRX/PRX* systems and *GST*s—was examined. The analysis revealed that the expression of antioxidative defense genes is dynamically modulated during ME induction, underscoring the importance of redox homeostasis in successful microspore reprogramming. Both cultivars shared a late (stages II–III) program with increased *SOD*s, selected *CAT/GPX* genes, rising *MDHAR*s, deployment of specific *TRX/GRX/PRX* members and broad *GST*s upregulation. Divergence emerged during progression: Igri showed a pronounced stage-III rise of *GR*s and targeted *TRX/GRX/PRX* transcripts, together with stronger activation of multiple *GST*s. When considered alongside diverse experimental data, these stage-restricted, cultivar-biased signatures support a hypothetical model in which strengthened ASC–GSH recycling and thiol-redox hubs sustain H_2_O_2_ signaling while limiting oxidative damage. Targeting *MDHAR*s, *GR*s, selected *TRX/GRX/PRX* genes, and *GST* subsets could improve ME efficiency and accelerate the integration of DH technology into modern crop breeding programs.

## 1 Introduction

Every living cell operates as a dynamic network of biochemical reactions governed by the cellular redox state. This balance—maintained through the production and removal of reactive oxygen, nitrogen, and sulfur species (ROS, RNS, and RSS)—is essential for homeostasis and underpins virtually all life processes. In plants, redox signals shape growth and development by modulating photosynthesis, respiration, and hormone signaling, as well as the activities of transcription factors and stress-related enzymes. Because plants are constantly exposed to environmental stress, rapid and accurate redox signalling is critical for survival. Redox cues have also been proposed as triggers of cellular reprogramming, including the shift from gametophytic to embryogenic development.

Our earlier studies revealed a central role for ROS in the induction of microspore reprogramming; whereby immature pollen grains are redirected towards embryogenic development (Żur et al., 2014a; Żur et al., 2021a). The resulting ELSs have the ability to regenerate into haploid and DH plants, highly valuable for basic research and breeding purposes. Microspores also represent an attractive target for genetic engineering, including genome editing, because modifications introduced into haploid cells can be stably fixed by chromosome doubling. However, the efficiency of ME is strongly genotype-dependent and often varies even between closely related genotypes (Krzewska et al., 2012; Żur et al., 2014a). Trial-and-error approaches to improve effectiveness of ME are therefore laborious, highlighting the need to better understand the underlying molecular mechanisms.

Advances in RNA sequencing (RNA-seq) have enabled detailed exploration of transcriptional networks regulating developmental reprogramming. Earlier studies provided insights into the transcriptomic changes associated with ME induction in barley (Bélanger et al., 2018; Bélanger et al., 2020). Building on this, our recent work compared two cultivars that differ strongly in embryogenic potential, providing a detailed map of the gene networks that orchestrate the shift in cellular machinery during ME induction (Nowicka et al., 2024). Based on our previous work in triticale (Żur et al., 2014a), we postulated a role for ROS in ME induction, a hypothesis supported by growing evidence. Tight control of redox homeostasis therefore appears to be a prerequisite for efficient microspore reprogramming. It is mediated by antioxidant and redox systems that regulate transfer of electrons from donor molecules to target proteins. Within this framework, antioxidant defense represents a key regulatory layer that scavenges reactive molecules and reduces oxidized substrates to protect microspores, while ROS themselves function as signaling molecules involved in growth, development, and stress adaptation. Recently published data highlight the complexity of the interactions among ROS, transcriptional and epigenetic regulators, plant hormones, metabolites, and suggest the potential mechanisms underlying ROS-mediated effects (Auverlot et al., 2024; Karpinska and Foyer, 2024; Lin et al., 2025).

**Figure 1:**
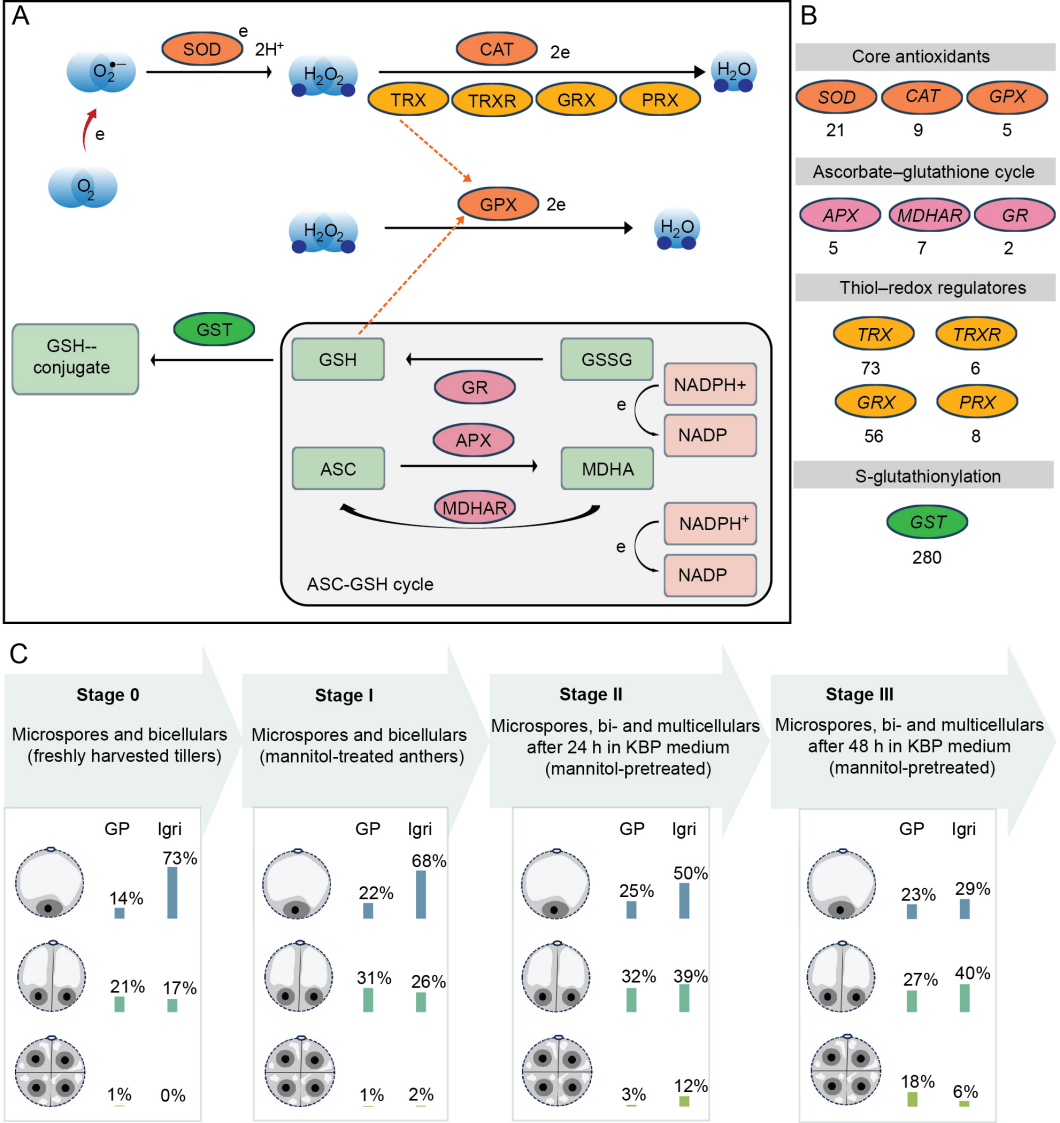
Antioxidant network and sampling stages during microspore embryogenesis. **(A)** Schematic of the ROS‐scavenging network surveyed in this study. Core antioxidants: SUPEROXIDE DISMUTASE (SOD), CATALASE (CAT) and GLUTATHIONE PEROXIDASE (GPX). Ascorbate–glutathione (ASC–GSH) cycle: ASCORBATE PEROXIDASE (APX), MONODEHYDROASCORBATE REDUCTASE (MDHAR) and GLUTATHIONE REDUCTASE (GR). Thiol–redox regulators: THIOREDOXIN (TRX), GLUTAREDOXIN (GRX), and PEROXIREDOXIN (PRX). S-glutathionylation is mediated by GLUTATHIONE S-TRANSFERASE (GST). **(B)** Gene families and numbers of annotated barley genes (HORVU.MOREX.r2). **(C)** Cytological composition across ME induction stages in two cultivars, Golden Promise (GP; recalcitrant) and Igri (responsive). Stage 0: microspores/bicellulars isolated from freshly harvested spikes. Stage I: isolates from anthers pre-treated 48 h in 0.4 M mannitol (21 °C). Stage II: isolates after mannitol-pretreatment followed by 24 h culture in KBP medium. Stage III: isolates after mannitol-pretreatment followed by 48 h in KBP. Icons with percentage bars indicate the proportions of uninucleate microspores, bicellular structures (after symmetric division) and multicellular structures (continued symmetric divisions). Detailed percentages and additional phenotypes are shown in **Supplementary Figure S1**. Quantitative data redrawn from (Nowicka et al., 2024).

Core antioxidant defenses include families of SUPEROXIDE DISMUTASES (SODs), CATALASES (CATs), and GLUTATHIONE PEROXIDASES (GPXs), which detoxify superoxide anion (O_2_^•–^) and hydrogen peroxide (H_2_O_2_) (**Figure 1A**) (Mittler, 2002). The electrons required for ROS reduction are supplied by metal ions, which serve as essential cofactors for enzymes such as SODs and CATs. In the case of peroxidases, which require electron donors, these electrons can be provided by REDUCED GLUTATHIONE (GSH), ASCORBATE (ASC) or THIOREDOXINS (TRXs), all of which also participate directly in redox reactions. Additional protection is provided by the ascorbate–glutathione cycle (ASC–GSH), where enzymes including MONODEHYDROASCORBATE REDUCTASE (MDHAR), DEHYDROASCORBATE REDUCTASE (DHAR), GLUTATHIONE REDUCTASE (GR), and ASCORBATE PEROXIDASE (APX) recycle oxidized forms of ASC (dehydroascorbic acid, DHA) and glutathione (glutathione disulfide, GSSG) (Foyer and Noctor, 2011). Glutathione also protects proteins against irreversible oxidation through S-glutathionylation, catalysed by GLUTATHIONE S-TRANSFERASES (GSTs). Many GSTs display also peroxidase activity (Noctor et al., 2024 and references therein). Other important electron transmitters include GLUTAREDOXINS (GRXs), PEROXIREDOXINS (PRXs) and TRXs which catalyze reversible disulfide bond formation and protect central metabolic pathways (Sevilla et al., 2023). Notably, the barley genome harbors multi-member gene families for every antioxidant class (**Figure 1B**) (Monat et al., 2019).

Here, we profile the transcriptional antioxidant network underpinning ME in barley by an extended analysis of previously published RNA-seq data from microspores and microspore-derived multicellular structures sampled across four early ME stages in two cultivars with contrasting embryogenic competence (Igri, responsive; Golden Promise, recalcitrant (Nowicka et al., 2024). We delineate antioxidant pathways, identify stage-specific markers of microspore reprogramming, and nominate candidate regulators whose expression distinguish the superior ME efficiency of Igri from Golden Promise. Consistent with this, we observe clear cultivar-dependent differences in antioxidant defense gene expression that likely contribute to divergent ME responsiveness and may tip the balance between successful reprogramming and stress-induced cell death. However, these conclusions rely on transcriptomic evidence, targeted functional assays will be required to determine whether reduced antioxidant activity is a primary driver of microspore mortality or instead reflects downstream consequences of broader metabolic and physiological reconfiguration during ME.

## 2 Materials and methods

### 2.1 Plant material, ME-induction stages, and RNA-seq sampling

This study builds on previously published RNA-seq data (Nowicka et al., 2024) from two barley (*Hordeum vulgare* L.) cultivars: Igri (winter type; HOR 10596; ME-responsive) and Golden Promise (spring type; HOR 16645; ME-recalcitrant). Detailed procedures for plant cultivation, ME induction, microspore isolation, and data processing are provided in Nowicka et al. (2024).

Four stages of ME-induction were analysed (**Figure 1C**):

Stage 0: Microspores isolated from freshly harvested tillers.

Stage I: Microspores isolated from anthers pre-treated with 0.4 M mannitol for 48 h at 21 °C.

Stages II–III: Microspores isolated from anthers cultured in KBP medium (Kumlehn et al., 2006) for 24 h (stage II) or 48 h (stage III) following the same mannitol pre-treatment.

Because Golden Promise is recalcitrant to ME induction, biochemical stimulation was provided by co-culture with immature pistils (+p) of wheat cultivar Bobwhite (Lippmann et al., 2015). Longitudinally bisected pistils (three halves per ml KBP medium) were added to isolated Golden Promise microspores, and stage II–III RNA-seq samples were collected after co-culture. Igri did not undergo co-culture. ME-induction efficiency and sample purity were assessed microscopically prior to RNA-seq (Daghma et al., 2014). Samples for RNA-seq were collected using a mannitol/maltose density-gradient method. The gradient was applied once for stage 0 and twice for stages I–III; stage III fractions were additionally sieve-filtered.

Bicellular pollen grains produced by asymmetric division were used as a gametophytic control. Pollen was isolated from freshly harvested tillers: cells were first enriched by a mannitol/maltose density gradient and then individually picked using a glass micropipette mounted on a micromanipulator under an inverted microscope, coupled to a microinjector for precise aspiration. Pollen RNA-seq data are unpublished (Kopeć et al., 2025).

### 2.2 RNA extraction, sequencing, and differential expression analysis

In brief, RNA-seq was performed with four biological replicates per ME induction stage and cultivar (32 samples in total) and three biological replicates per cultivar for bicellular pollen (6 samples). Total RNA was extracted using the NucleoSpin RNA Plant Kit (740949.50; Macherey-Nagel, Düren, Germany). Samples with RNA integrity values (RIN) > 6.0 were used to prepare poly(A)-selected mRNA libraries (NEBNext Ultra™ RNA Library Prep Kit for Illumina). Libraries were sequenced as 150-bp paired-end reads on an Illumina NovaSeq platform (Genewiz). Raw reads were adapter-trimmed using Trim Galore (v0.4.1) and aligned to the *H. vulgare* cultivar Morex reference genome v2 (Monat et al., 2019) using HISAT2 (v2.1.0). Gene-level read counts were obtained with Subread/featureCounts (v1.5.2) using the corresponding genome annotation. Differential expression analysis was performed on raw count data using DESeq2 (v1.24.0) in R (v3.6.3) with the Wald test under a negative binomial model. *P*-values were adjusted for multiple testing using the Benjamini–Hochberg procedure, and genes with FDR < 0.05 were considered differentially expressed. Transcript abundance was reported as FPKM, and log_2_ fold changes (log_2_FC) were calculated by DESeq2.

The RNA-seq dataset is publicly available in the NCBI Gene Expression Omnibus (GSE233486).

### 2.3 Antioxidant gene selection and classification

To investigate redox‐associated transcriptional responses during ME induction, we have completed the RNA-seq dataset with a targeted set of antioxidant and redox-related genes. Candidate functions were curated manually from biochemical pathway knowledge and primary literature (Mittler, 2002; Foyer and Noctor, 2011; Sevilla et al., 2023). In total, 472 genes (**Figure 1B**) were curated and assigned to four functional categories:

i) core antioxidant genes: *SUPEROXIDE DISMUTASES* (*SOD*s), *CATALASES* (*CAT*s), *GLUTATHIONE PEROXIDASES* (*GPX*s);

ii) ASC–GSH cycle genes: *ASCORBATE PEROXIDASES* (*APX*s), *MONODEHYDROASCORBATE REDUCTASES* (*MDHAR*s), *DEHYDROASCORBATE REDUCTASES* (*DHAR*s) and *GLUTATHIONE REDUCTASE* (*GR*s);

iii) redox-regulated thiol genes: *THIOREDOXINS (TRX*s*), THIOREDOXIN REDUCTASE* (*TRXR*s), *GLUTAREDOXINS (GRX*s*), PEROXIREDOXINS (PRX*s);

iv) genes involved in S-glutathionylation: *GLUTATHIONE S-TRANSFERASES* (*GST*s).

Candidate genes were initially retrieved from the *H. vulgare* Morex v2 annotation (Monat et al., 2019) using keyword- and function-based searches. When gene families were incompletely annotated or ambiguous, orthologous *Arabidopsis thaliana* protein sequences were used as queries for manual homology searches in EnsemblPlants to identify the corresponding barley genes.

### 2.4 Marker gene criteria and cross-study validation

Stage-specific markers were genes that, at a given induction stage, met all of the following criteria: (i) FPKM > 10 in both cultivars; (ii) no significant cultivar effect at that stage (FDR-adjusted *P* > 0.05); (iii) log_2_FC > 2 relative to the subsequent stages; and (iv) absent or very low expression in the gametophytic control (bicellular pollen, P). Cultivar-specific markers were genes that, at a given stage, met: (i) FPKM > 5; (ii) a significant difference between cultivars (FDR-adjusted *P* < 0.05); (iii) log_2_FC > 2 relative to the second cultivar at the same stage; and (iv) absent or very low expression in P. Genes that did not meet these thresholds but showed directionally consistent patterns are reported as trend-level candidates for stage or cultivar specificity.

For cross-study validation, thiol-related and *GST* genes reported for the ME-responsive cultivar Gobernadora (Bélanger et al., 2018), originally annotated against the Morex v1 (Mascher et al., 2017) reference genome, were mapped to Morex v2 identifiers using reciprocal BLASTP searches using EnsemblPlants database.

### 2.5 Data analysis and visualization

Gene filtering, selection, and summarization were performed in R (v4.2.2) using custom scripts. Heatmaps were generated with Heatmapper (http://heatmapper.ca/expression/). Matrix bubble charts and additional visualizations were produced using ggplot2.

## 3 Results

### 3.1 Cytological composition across ME-induction stages

Using the published dataset of Nowicka et al. (2024), we assessed oxidative-stress-related transcriptomic responses across four ME-induction stages in two barley cultivars, Igri (responsive) and Golden Promise (recalcitrant): stage 0 (isolation from freshly harvested tillers), stage I (48 h in 0.4 M mannitol at 21 °C), and stages II–III (24 h and 48 h after transfer to KBP, respectively). Each stage comprised mixed cell populations with cultivar-specific composition (**Figure 1C**; **Supplementary Figure S1**). At stage 0, Igri was enriched for uninucleate microspores (73%) with fewer bicellular structures after symmetric division (17%). Golden Promise contained fewer uninucleates (14%) and more bicellulars (21%) whereas a high proportion of microspores remained unidentified. Stage I showed comparable reprogramming (≈30% symmetric divisions), but uninucleates remained more frequent in Igri (68%) than in Golden Promise (22%). Across stages II–III, Golden Promise maintained ≈30% bicellular structures, while multicellular structures—arising from continued symmetric divisions—increased from 3% at stage II to 18% at stage III. In Igri, bicellulars remained ≈40% across stages II–III, with multicellular structures at 12% in stage II and 6% in stage III. These differences were more pronounced in cultures lacking the stimulatory effect of co-cultured pistils from the highly embryogenic wheat cultivar Bobwhite (−p; **Figure 1C**; **Supplementary Figure S1**). This staged cytology provides the framework for the antioxidant/redox transcriptomic analyses that follow.

### 3.2 Core antioxidant enzymes were reprogrammed in a stage- and cultivar-dependent manner

**Figure 2:**
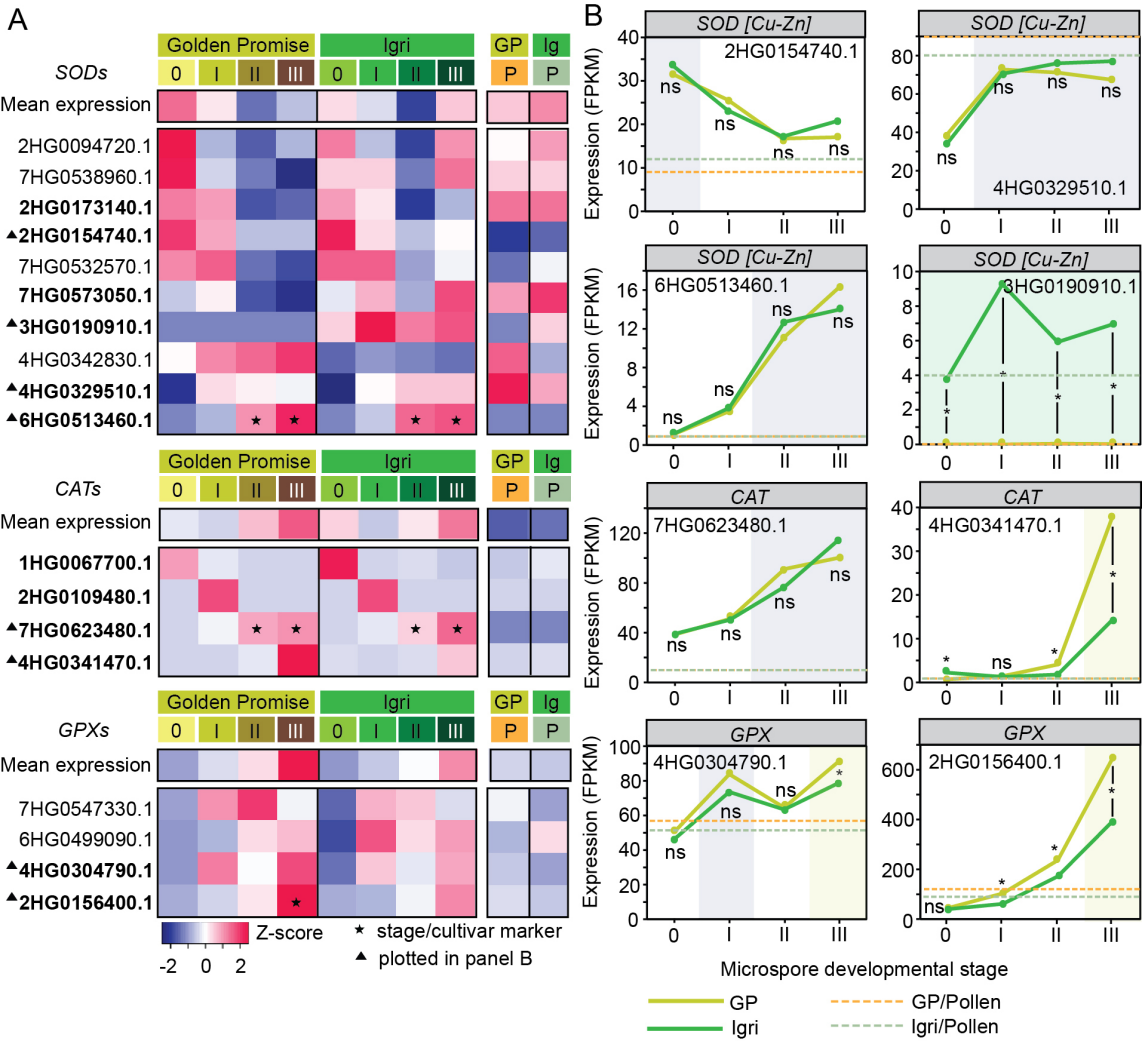
Transcriptional dynamics of genes encoding core antioxidant enzymes during early microspore embryogenesis in barley. **(A)** Heatmaps showing stage- and cultivar-specific expression patterns of representative *SUPEROXIDE DISMUTASE* (*SOD*; n = 10 of 15 transcriptionally active), *CATALASE* (*CA*s; n = 4 of 9 transcriptionally active), and *GLUTATHIONE PEROXIDASE* (*GPX*; n = 4 of 5 transcriptionally active) genes in Golden Promise (GP) and Igri. Expression values are shown as row-wise Z-scores; the top row in each block represents the mean expression of all detected members of the corresponding gene family. Only genes with detectable expression (FPKM > 0) in at least one stage in either cultivar were included. Stages 0–III represent successive steps of ME induction, whereas P denotes bicellular pollen (gametophytic development). Gene identifiers are abbreviated by omitting the common HORVU.MOREX.r2 prefix. ▲ indicates genes plotted in **(B)**; bold gene IDs denote genes discussed in the text. ★ indicates marker genes, defined as transcripts exhibiting stage-specific and/or cultivar-specific expression during ME induction. **(B)** Expression trajectories for selected *SOD*, *CAT* and *GPX* genes across ME stages. Asterisks indicate between-cultivar differences at a given stage (DESeq2, FDR-adjusted P < 0.05); ‘ns’ indicates non-significance. Background shading indicates genes classified as stage-specific (grey), Igri-specific (green), or Golden Promise-specific (yellow). Dashed horizontal lines indicate the corresponding pollen expression level for each cultivar. Additional information on these gene families is provided in **Supplementary Figure S2**.

We first profiled genes encoding the core antioxidant enzymes that constitute the primary defense against ROS — *SOD*s, *CAT*s and *GPX*s (**Figure 2**, **Supplementary Figure S2**). Of 21 annotated *SOD* genes, 15 were transcriptionally active (FPKM > 0) in at least one induction stage, together with five of nine *CAT*s and all five *GPX*s (**Supplementary Figure S2A**). *SOD*s spanned a wide expression range: in Golden Promise, most transcripts were moderate (10–100 FPKM) with few highly expressed (>100 FPKM), whereas in Igri expression was more evenly split between low (1–10 FPKM) and moderate levels. *CAT*s were generally weakly expressed in both cultivars, while *GPX*s were consistently stronger, predominantly at moderate–high levels (**Supplementary Figure S2B**). Most expressed *SOD*s belonged to *Cu/Zn-SOD* orthogroups (Arabidopsis *CSD1/2/3*), with fewer chloroplastic *Fe-SOD*s and a single mitochondrial *MnSOD*. Two *CAT*s aligned with *CAT2* and several *GPX*s with *GPX1/6* (**Supplementary Dataset 1**).

Family-level averages revealed distinct trends (**Figure 2A**, **Supplementary Figure S2C**). Mean *SOD* expression ranged from ~40 FPKM (Igri, stage II) to ~60 FPKM (Golden Promise, stage I), with maxima at stage 0 in Golden Promise and stage III in Igri; bicellular pollen (P; gametophytic control) showed *SOD* levels comparable to induction stages. *CAT*s averaged 12–28 FPKM and peaked at stage III in both cultivars. *GPX*s exceeded *SOD*s and *CAT*s overall, tended to be higher in Golden Promise, and peaked at stage III (~180 FPKM). In contrast to induction stages, *CAT* and *GPX* transcripts were low in mature pollen.

Gene-level profiles highlighted discrete regulatory modes (**Figures 2A, B**). Among *SOD*s, some transcripts were induced early (stages 0–I; e.g. *MnSOD* HORVU.MOREX.r2.2HG0173140.1; *Cu/Zn-SOD* HORVU.MOREX.r2.2HG0154740.1), whereas others rose later (stages I–III; e.g. *Cu/Zn-SOD*s HORVU.MOREX.r2.4HG0329510.1 and HORVU.MOREX.r2.6HG0513460.1—the latter strongly upregulated in both cultivars at stages II–III with FPKM > 10 but not detected in mature pollen, supporting its candidacy as an ME-stage marker). Additional cultivar specificity was evident: *Cu/Zn-SOD* HORVU.MOREX.r2.7HG0573050.1 was moderate at stages 0–II in both cultivars but became Igri-specific at stage III, whereas HORVU.MOREX.r2.3HG0190910.1 was confined to Igri.

Three *CAT*s showed clear stage specificity: HORVU.MOREX.r2.1HG0067700.1 at stage 0 (uninucleate microspores), HORVU.MOREX.r2.2HG0109480.1 at stage I (osmotic treatment), and HORVU.MOREX.r2.4HG0341470.1 at stage III, with higher expression in Golden Promise. Another *CAT* (HORVU.MOREX.r2.7HG0623480.1) increased progressively across Stages II–III in both cultivars and served as an II–III marker. All *CAT*s were very low in mature pollen (**Figures 2A, B**).

*GPX*s also exhibited stage- and cultivar-dependent regulation: HORVU.MOREX.r2.4HG0304790.1 peaked at stages I and III, and HORVU.MOREX.r2.2HG0156400.1 marked Golden Promise at stage III. As with *CAT*s, *GPX* transcripts were low in mature pollen (**Figures 2A, B**).

Collectively, core antioxidant genes show dynamic regulation during ME, with stage- and cultivar-specific patterns that distinguish the responsive Igri from the recalcitrant Golden Promise.

### 3.3 ASC–GSH cycle genes showed late MDHAR rise and stronger GR induction in responsive cultivar

**Figure 3:**
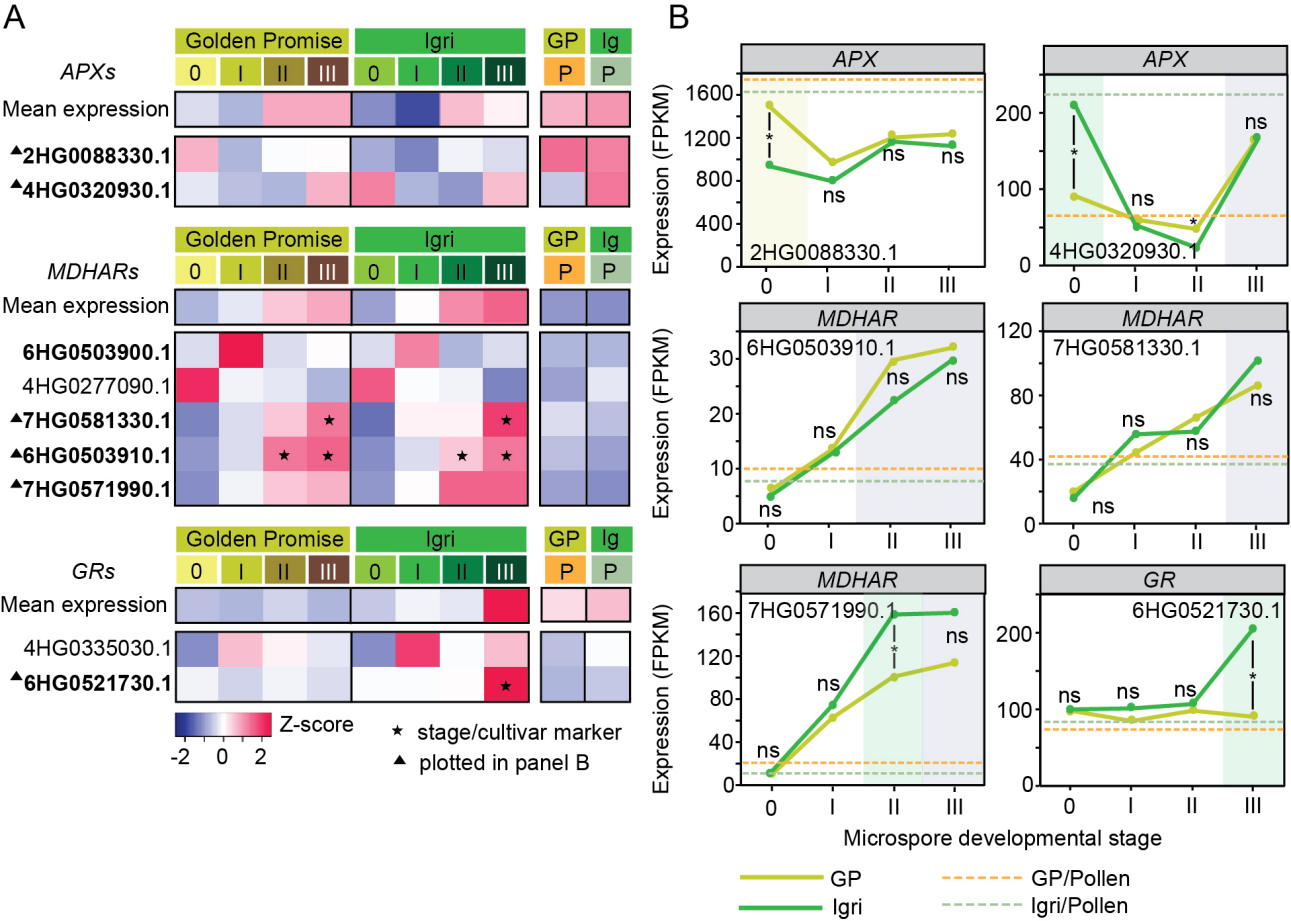
Transcriptional dynamics of ascorbate–glutathione (ASC–GSH) cycle genes during early microspore embryogenesis in barley. **(A)** Heatmaps of representative *ASCORBATE PEROXIDASE* (*APX*; n = 2 out of 4 transcriptionally active), *MONODEHYDROASCORBATE REDUCTASE* (*MDHAR*; n = 5, all transcriptionally active) and *GLUTATHIONE REDUCTASE* (*GR*; n=2, all transcriptionally active) genes showing stage- and cultivar-specific expression patterns (row Z-scores) in Golden Promise (GP) and Igri. The top row in each block indicates the mean expression of all detected members of the corresponding gene family. Only genes with detectable expression (FPKM > 0) in at least one stage in either cultivar were included. Stages 0–III represent successive steps of ME induction, whereas P denotes bicellular pollen (gametophytic development). Gene IDs are abbreviated by omitting the common HORVU.MOREX.r2 prefix. ▲ indicates genes plotted in **(B)**; bold gene IDs denote genes discussed in the text. ★ indicates marker genes, defined as transcripts exhibiting stage-specific and/or cultivar-specific expression during ME induction. **(B)** Expression trajectories for selected *APX*, *MDHAR* and *GR* genes across ME stages. Asterisks indicate between-cultivar differences at a given stage (DESeq2, FDR-adjusted P<0.05); ‘ns’ indicates non-significance. Background shading indicates genes classified as stage-specific (grey), Igri-enriched (green) or Golden Promise-enriched (yellow). Dashed horizontal lines indicate the corresponding pollen expression level for each cultivar. Additional family-level expression summaries are provided in **Supplementary Figure S3**.

We next analyzed *APX*, *MDHAR*, and *GR* families (**Figure 3**, **Supplementary Figure S3**). A distinct *DHAR* gene family is not annotated in the barley Morex v2 genome; homology-based searches indicated that DHAR-like functions are represented by GST-annotated genes (**Supplementary Dataset 1**). Most genes were expressed (*APX*: 4/5; *MDHA*R: 5/7; *GR*: 2/2; 14 total; **Supplementary Figure S3A**), mapping to orthogroups containing the corresponding Arabidopsis genes (**Supplementary Dataset 1**).

At the family level, *APX* genes exhibited the highest mean expression among all antioxidant gene families, maintaining consistently high transcript abundance across induction stages and in pollen in both cultivars (≈300–500 mean FPKM). Notably, a single *APX* transcript (HORVU.MOREX.r2.2HG0088330.1) displayed very high and stable expression across all ME stages and in pollen, reaching ≈1000 FPKM (**Figures 3A, B**, **Supplementary Figure S3B**). In contrast, *MDHAR*s increased progressively from stage 0 to stage III, particularly in Igri (up to ~77 FPKM), with pollen lower than induction stages. *GR*s were expressed at moderate levels (~60 FPKM) across ME stages and in pollen, except for a marked rise in Igri at stage III (~115 FPKM).

Gene-specific patterns reinforced these trends (**Figures 3A, B**). Within *APX*, HORVU.MOREX.r2.4HG0320930.1 was high in Igri at stage 0 and re-emerged at stage III, showing stage-III specificity in both cultivars. Several *MDHAR*s (HORVU.MOREX.r2.6HG0503910.1, HORVU.MOREX.r2.7HG0581330.1, HORVU.MOREX.r2.7HG0571990.1) showed progressive induction marking stages II–III, whereas HORVU.MOREX.r2.6HG0503900.1 peaked at stage I in both cultivars. Among *GR*s, HORVU.MOREX.r2.6HG0521730.1 emerged as a stage III–specific marker of Igri. In mature pollen, *APX*s expression was high in one or both cultivars, whereas *MDHAR*s and *GR*s were low.

In summary, *APX* transcripts are abundant but largely stage-stable, *MDHAR*s rise with ME progression, and *GR* showed an Igri-biased stage-III induction.

### 3.4 Thiol-redox regulators showed late activation with responsive cultivar enrichment

**Figure 4:**
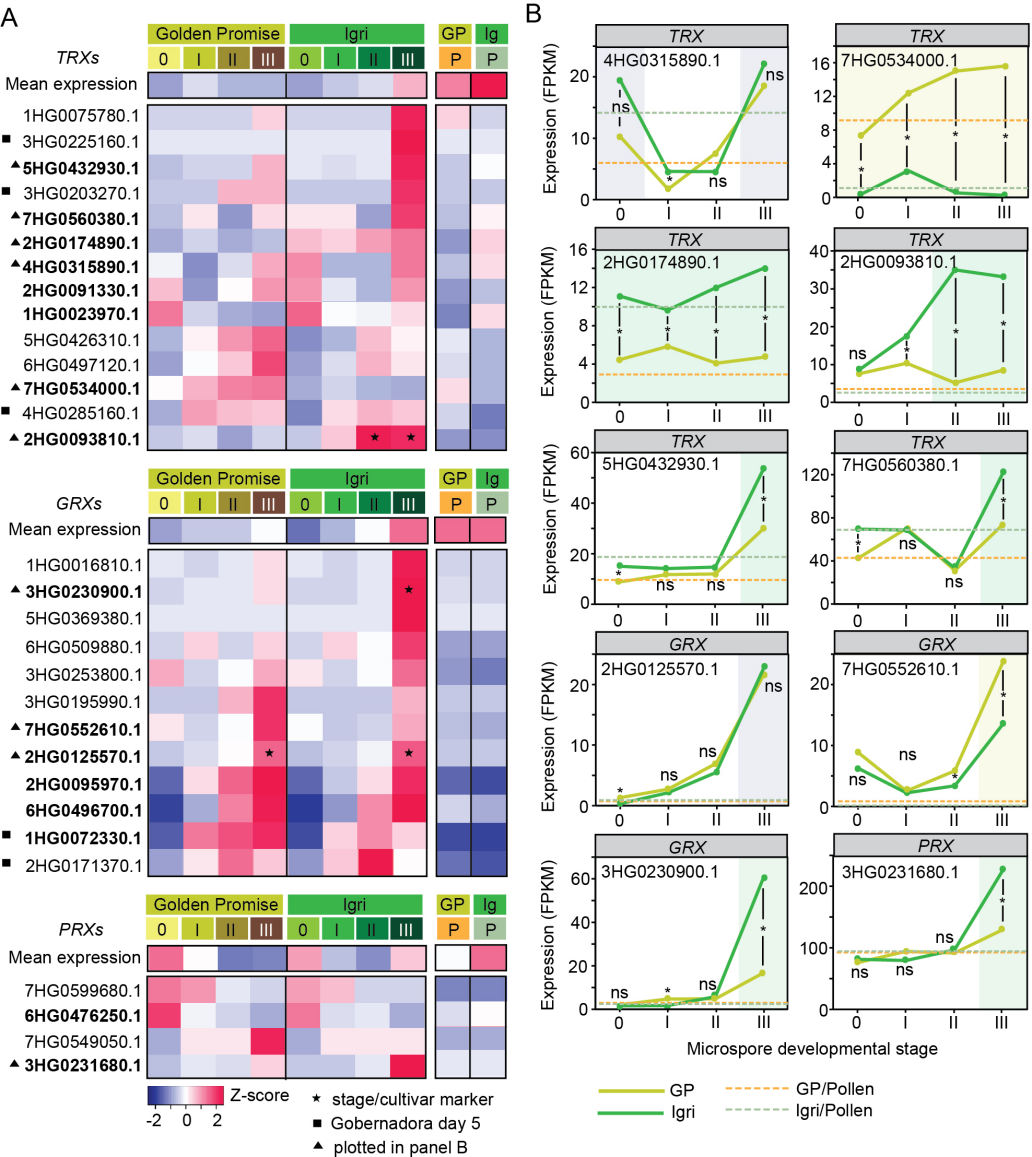
Transcriptional dynamics of thiol–redox regulatory genes during early microspore embryogenesis in barley. **(A)** Heatmaps of representative *THIOREDOXIN* (*TRX*; n = 14 out of 57 transcriptionally active), *GLUTAREDOXIN* (*GRX*; n = 12 out of 37 transcriptionally active) and *PEROXIREDOXIN* (*PRX*; n = 4 out of 8 transcriptionally active) genes showing stage- and cultivar-specific expression patterns (row Z-scores) in Golden Promise (GP) and Igri. The top row in each block indicates the mean expression of all detected members of the corresponding gene family. Only genes with detectable expression (FPKM > 0) in at least one stage in either cultivar were included. Stages 0–III represent successive steps of ME induction, whereas P denotes bicellular pollen (gametophytic development). Symbols next to gene IDs denote: bold gene IDs genes discussed in the text. ★ indicates marker genes, defined as transcripts exhibiting stage-specific and/or cultivar-specific expression during ME induction. ▲ indicates genes plotted in **(B)**. ■ marks genes reported as upregulated at day 5 of culture in the ME-responsive cultivar Gobernadora (Bélanger et al., 2018). Detailed data are provided in Dataset 1. **(B)** Expression trajectories for selected *TRX*, *GRX*, and *PRX* genes across ME stages. Asterisks indicate between-cultivar differences at a given stage (DESeq2, FDR-adjusted P<0.05); ‘ns’ indicates non-significance. Shading indicates the stage where each gene is stage-specific (grey), Igri-specific (green), or Golden Promise-specific (yellow). Dashed horizontal lines indicate the pollen expression level for each cultivar. Additional family-level expression summaries for *TRX*s/*GRX*s/*PRX*s and *THIOREDOXIN REDUCTASE*s (*TRXR*s) are provided in **Supplementary Figure S4**.

We examined also the transcriptional activity of thiol–redox regulatory families (**Figure 4**, **Supplementary Figure S4**). Of 143 annotated genes, most were expressed in at least one stage: 57/63 *TRX*s, 37/56 *GRX*s, all 6 *TRXR*s and all 8 *PRX*s (**Supplementary Figure S4A**). *TRX* and *GRX* transcripts generally accumulated at low–to–moderate levels, with few highly expressed members; *TRXR* and *PRX* showed similar distributions (**Supplementary Figure S4B**).

At the family level, mean expression was relatively stable across stages 0–III and in pollen, with only minor cultivar differences (**Figure 4A**, **Supplementary Figure S4C**). *TRXR* was notably invariant: all six members showed no clear induction with ME or genotype effects (**Supplementary Dataset 1**).

In contrast, several *TRX*s and *GRX*s were selectively mobilized at later stages (II–III), particularly in Igri. Representative trajectories illustrated these contrasts (**Figures 4A, B**). For example, *TRX*s HORVU.MOREX.r2.1HG0023970.1, HORVU.MOREX.r2.4HG0315890.1, and HORVU.MOREX.r2.2HG0091330.1 exhibited biphasic dynamics: high at stage 0, decreased at stages I–II, then re-induced at stage III—Igri-specific for HORVU.MOREX.r2.1HG0023970.1, shared by both cultivars for the other two. Strong cultivar biases were evident: HORVU.MOREX.r2.7HG0534000.1 was consistently higher in Golden Promise, whereas HORVU.MOREX.r2.2HG0174890.1 was higher in Igri across stages. Marker-like behavior included Igri-specific induction at stages II–III (HORVU.MOREX.r2.2HG0093810.1) and stage-III-restricted increases (HORVU.MOREX.r2.5HG0432930.1, HORVU.MOREX.r2.7HG0560380.1). Among *GRX*s, HORVU.MOREX.r2.2HG0125570.1 acted as a stage-III marker in both cultivars, HORVU.MOREX.r2.7HG0552610.1 was enriched in Golden Promise at stage III, and HORVU.MOREX.r2.3HG0230900.1 was Igri-stage III marker. Several transcripts (HORVU.MOREX.r2.6HG0496700.1, HORVU.MOREX.r2.2HG0095970.1, HORVU.MOREX.r2.1HG0072330.1) accumulated progressively across induction. Within *PRX*s, HORVU.MOREX.r2.6HG0476250.1 was stage-I specific in both cultivars, whereas HORVU.MOREX.r2.3HG0231680.1 showed clear Igri specificity at stage III.

To validate these patterns, a cross-study comparison was performed with the ME-responsive cultivar Gobernadora, in which three *TRX* and two *GRX* genes were reported as upregulated at day 5 of culture (Bélanger et al., 2018). The expression profiles of these candidates were therefore examined in the Igri and Golden Promise datasets. Two *TRXs* showed Igri stage III-specific accumulation, whereas the third was induced in both cultivars across stages I–III; similarly, both *GRXs* displayed increased expression in both cultivars across stages I–III (**Figure 4A**, **Supplementary Dataset 1**).

Notably, a subset of *TRX*, *GRX* and *PRX* genes was not ME-responsive but was strongly expressed in mature pollen, indicating developmental rather than reprogramming regulation (**Supplementary Figures S4D, E**).

Hence, although family-level expression appears stable, distinct *TRX*/*GRX*/*PRX* members are selectively deployed in a stage- and cultivar-dependent manner, with late (II–III) induction particularly prominent in Igri.

### 3.5 Enhanced GST activation at stage III of ME in the responsive barley cultivar

**Figure 5:**
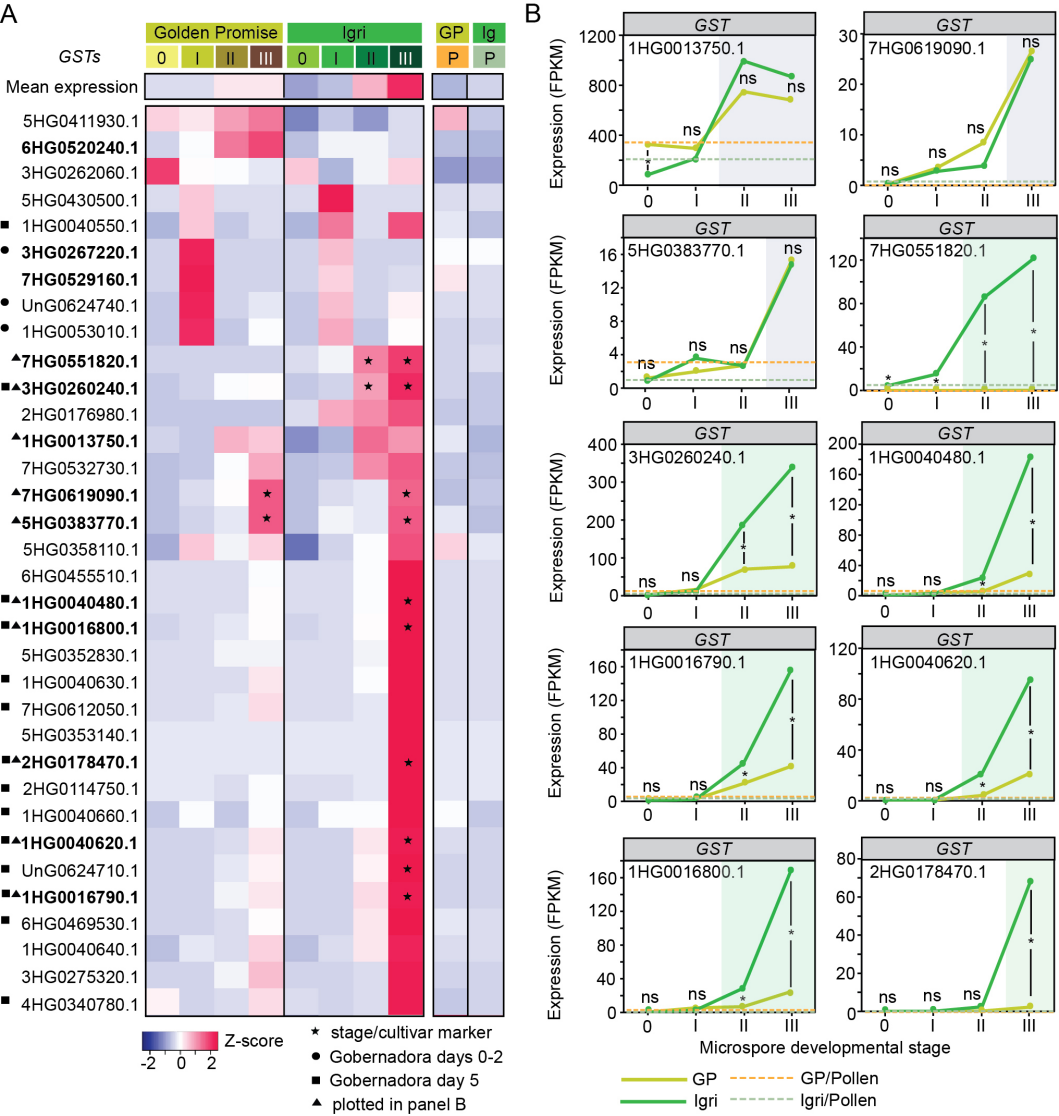
Transcriptional dynamics of *GLUTATHIONE S-TRANSFERASE* (*GST*) genes during early microspore embryogenesis in barley. **(A)** Heatmap of 34 representative *GST* genes (out of 180 transcriptionally active) showing stage- and cultivar-dependent expression patterns in Golden Promise (GP) and Igri. Expression is displayed as row Z-scores; the top row indicates the mean expression of all detected *GST*s. Only genes with detectable expression (FPKM > 0) in at least one stage in either cultivar were included. Stages 0–III represent successive steps of ME induction, whereas P denotes bicellular pollen (gametophytic development). Gene IDs are abbreviated by omitting the common HORVU.MOREX.r2 prefix. Symbols next to gene IDs denote: ▲ genes plotted in panel **(B)**; ● *GST*s reported as upregulated in the ME-responsive cultivar Gobernadora at days 0–2 of culture; ■ *GST*s reported as upregulated in Gobernadora at day 5 (Bélanger et al., 2018). Bold gene IDs indicate genes specifically discussed in the main text. Extended data are provided in Dataset 1 and in **Supplementary Figure S5**. **(B)** Expression trajectories (FPKM) for selected *GST*s across ME stages in GP and Igri. Asterisks indicate significant between-cultivar differences at a given stage (DESeq2; FDR-adjusted P < 0.05); ns denotes non-significance. Background shading indicates genes classified as stage-specific (grey) or Igri-enriched (green). Dashed horizontal lines indicate the corresponding pollen expression level for each cultivar. Additional family-level expression summaries are provided in **Supplementary Figure S5**.

To assess the role of GST during ME induction, we profiled the expression of 280 annotated *GST* genes (**Figure 5**, **Supplementary Figure S5**). Exactly half of these genes (140/280) were transcriptionally active (FPKM > 0) in at least one developmental stage in either cultivar (**Supplementary Figure S5A**). The expressed *GST* genes are distributed across various classes, with a particularly strong representation of the theta class (*GSTT3, n = 41*, **Supplementary Dataset 1**). Most transcripts accumulated at very low to low levels (0–10 FPKM), with relatively few reaching moderate (10–100 FPKM) or high (≥100 FPKM) abundance. Notably, the responsive cultivar Igri contained more strongly expressed *GST*s at stage III than Golden Promise (**Supplementary Figure S5B**).

Mean *GST* expression increased progressively from stage 0 to stage III in both cultivars, peaking in Igri at stage III (~40 FPKM). Pollen (P) showed low *GST* expression, comparable to stage 0 (**Figure 5A**, **Supplementary Figure S5C**).

Closer inspection revealed diverse expression trajectories (**Figures 5A, B**). Several transcripts were preferentially induced in Golden Promise at stage I (e.g. HORVU.MOREX.r2.7HG0529160.1, HORVU.MOREX.r2.3HG0267220.1) or at stages II–III (e.g. HORVU.MOREX.r2.6HG0520240.1). HORVU.MOREX.r2.1HG0013750.1 was the most highly expressed *GST* overall, with strong accumulation at stages II and III in both cultivars. Additional common stage markers included genes specifically induced at stage III (e.g. HORVU.MOREX.r2.7HG0619090.1, HORVU.MOREX.r2.5HG0383770.1). A substantial subset was enriched in Igri at stages II–III, with maxima at stage III: some maintained high expression across both stages (e.g. HORVU.MOREX.r2.7HG0551820.1, HORVU.MOREX.r2.3HG0260240.1), whereas others showed a stepwise rise from moderate (stage II) to strong (stage III) levels (e.g. HORVU.MOREX.r2.1HG0040480.1, HORVU.MOREX.r2.1HG0016790.1, HORVU.MOREX.r2.1HG0040620.1, HORVU.MOREX.r2.1HG0016800.1). A final group acted as Igri-specific stage-III markers (e.g. HORVU.MOREX.r2.2HG0178470.1).

Similarly, cross-study comparison with the ME-responsive cultivar Gobernadora supported the stage specificity observed here (Bélanger et al., 2018). Seven *GST*s reported as upregulated at days 0–2 of culture were examined in the present dataset and were found to be associated primarily with early induction: three were upregulated at stage I in both cultivars but accumulated more strongly in Golden Promise, whereas four showed elevated expression across stages 0–II; in all cases, expression declined at stage III. In contrast, of 19 *GST*s reported as upregulated at day 5, thirteen displayed clear Igri stage III-enrichment, including seven genes with strict marker-like behavior. This concordance was taken as independent validation of the dataset and supported the association of late *GST* activation with responsive genotypes (**Figure 5A**, **Supplementary Figure S5D**, **Supplementary Dataset 1**).

Together, these results indicate that *GST* activity intensifies during the transition to embryogenesis, with a pronounced stage-III induction in the responsive cultivar Igri.

## 4 Discussion

Studies in triticale and barley have shown that ME induction is accompanied by the accumulation of ROS, which act as key determinants of microspore fate—promoting embryogenic reprogramming at moderate levels but triggering cell death when excessive (Żur et al., 2008; Żur et al., 2009; Żur et al., 2014a; Żur et al., 2019; Żur et al., 2021a; Żur et al., 2021b). To identify antioxidant components that sustain a pro-embryogenic redox balance, we conducted an *in silico* analysis of genes encoding major antioxidant and redox enzymes in two barley cultivars with contrasting embryogenic potential. Despite well-documented differences in ME responsiveness (Kumlehn et al., 2006; Żur et al., 2021a), cytological analysis showed that both cultivars initiated microspore reprogramming with comparable efficiency at stage I and displayed a broadly similar profile of antioxidant gene expression at this early step. Divergence became apparent 24 h after transfer to induction medium. It was most pronounced at 48 h, when the number of differentially expressed genes peaked. These observations support a model in which an initial, shared activation of antioxidant genes enables survival and entry into reprogramming, whereas cultivar-specific transcriptional programs emerging later contribute to differential embryogenic competence.

### 4.1 Elements of antioxidative defense identified in barley microspores and activated during ME induction

Our survey revealed a broad antioxidant repertoire in barley microspores: 35 core antioxidant genes, 14 ASC–GSH cycle genes, 143 *TRX*/*GRX/PRX* genes, and a large *GST* family (280 members). A similarly large set of 330 *TaGST*s was reported in wheat (Wang et al., 2019). Approximately 80% of these genes were transcriptionally active during ME induction. Significant expression changes were detected for nearly 80% of core antioxidant and *TRX*/*GRX* genes, ~90% of ASC–GSH genes, and ~70% of *GST*s—evidence of extensive redox rewiring during induction.

### 4.2 The dual role of core antioxidative enzymes in microspore protection and differentiation

Among core enzymes, *SOD*s emerged as prominent candidates. Most annotated *SOD*s corresponded to Arabidopsis Cu/Zn isoforms localized to the cytosol, chloroplasts, or peroxisome. These isoforms were also the most abundant in anthers of triticale subjected to ME-inducing cold pre-treatment (Żur et al., 2014a). They are known to function in development and respond strongly to temperature, drought, and salinity (Gill and Tuteja, 2010; Wang et al., 2016; Li et al., 2017). Genotype-biased differences were clearest at stage III, where several *SOD* transcripts (e.g. HORVU.MOREX.r2.7HG0573050.1) accumulated more strongly in the responsive cultivar Igri. Similarly, increased accumulation of *SOD* transcripts was observed in *Brassica napus* microspores redirected towards ME (Rueda-Varela et al., 2025).

Functionally, SODs catalyze the dismutation of O_2_^•–^ to H_2_O_2_, thereby limiting the formation of highly reactive ^•^OH via the Haber–Weiss reaction (Mittler, 2002). Being less reactive, H_2_O_2_ acts as a signaling molecule that modulates gene expression and enzyme activity through cysteine oxidation-based redox regulation (Souza, 2025). Our previous work supports a role for H_2_O_2_ as a trigger for ME as excessive H_2_O_2_ elimination improved microspore survival but reduced reprogramming efficiency (Żur et al., 2014a; Żur et al., 2021a). It suggests that maintaining a signaling-competent H_2_O_2_ pool is essential for successful ME induction. However, subsequent studies in triticale and barley demonstrated that both O_2_^•–^ and H_2_O_2_ accumulate in the microspore cytoplasm after ME-inducing treatment (Żur et al., 2021b). Notably, enhanced generation of O_2_^•–^ was detected in proximity to the nuclei, pointing to its potential role in early signaling events. This interpretation is consistent with recent insights summarized by Karpinska and Foyer (2024), who highlight a previously underappreciated function of superoxide in compartment-specific signaling within the plant cell nucleus.

Consistently, *CAT*s showed stage-specific expression—distinct isoforms predominating at different phases—whereas many *GPX*s (with higher affinity for H_2_O_2_ and frequently implicated in signaling) were induced by mannitol and further upregulated at 48 h of *in vitro* culture. Higher *CAT* and *GPX* transcript abundance in Golden Promise (e.g. *CAT* HORVU.MOREX.r2.4HG0341470.1; *GPX* HORVU.MOREX.r2.2HG0156400.1) may reflect over-scavenging that dampens H_2_O_2_ signaling required for the developmental switch, consistent with the negative non-linear relationship between *CAT* activity and ME efficiency reported in triticale (Żur et al., 2014a).

### 4.3 Functional divergence of ascorbate and glutathione during ME induction

Low-molecular-weight antioxidants ASC and GSH are abundant and widely distributed in plant cells. They directly detoxify ROS, protecting cell components from oxidative damages and help maintain the cellular redox environment required for effective metabolism and development. Their oxidized forms (MDHA, DHA, GSSG) are efficiently recycled in the ASC–GSH cycle due to concerted action of reducing enzymes (MDHAR, DHAR and GR; Foyer and Noctor, 2011). Two observations stood out. First, several *MDHAR* transcripts increased progressively during induction—either similarly in both cultivars or with a stronger response in Igri (e.g. HORVU.MOREX.r2.6HG0503910.1 and HORVU.MOREX.r2.7HG0581330.1)—consistent with sustained ASC recycling during reprogramming. Second, one *GR* (HORVU.MOREX.r2.6HG0521730.1) showed a cultivar-biased induction at stage III, consistent with prior evidence that elevated *GR* activity is associated with microspore competence for ME (Żur et al., 2021b). In contrast, *APX* transcripts were highly abundant but largely stage stable in both cultivars, suggesting a constitutive role in maintaining basal H_2_O_2_ detoxification during both gametophytic and embryogenic development rather than driving stage-specific transitions.

Together, these patterns support a framework in which ASC-dependent redox buffering primarily supports microspore and pollen survival in both cultivars, whereas efficient GSH recycling becomes especially important in rapidly dividing, embryogenic cells of Igri. Supporting this view, a recent report showed that a local GSH burst in wounded Arabidopsis roots shortens G1 to accelerate division and regeneration (Lee et al., 2025), offering a plausible mechanistic link. Consistently, exogenous GSH in our earlier works maintained microspore viability and stimulated ELS development (Żur et al., 2019; Żur et al., 2021b).

### 4.4 Cumulative activity of thiol reductases as underestimated element of successful microspore reprogramming

Thiol oxidoreductases further integrate redox control with development. *TRXs* and *GRXs*—abundant and broadly localized—regulate protein redox state, reduce disulphide and mixed disulphide bonds using ferredoxin/NADPH (TRX systems) or GSH (GRX), and can donate electrons to other antioxidant enzymes (Meyer et al., 2009; Meyer et al., 2012; Sevilla et al., 2023). They also participate in retrograde communication that coordinates gene expression during stress acclimation (Sevilla et al., 2023). We observed selective late (stages II–III) induction of specific *TRX* and *GRX* members, particularly in Igri, consistent with roles in supporting rapid divisions and stabilizing redox-sensitive enzymes during multicellular structures formation. Notably, this cultivar-biased late activation was independently supported by comparison with the ME-responsive cultivar Gobernadora, in which several of the same thiol–redox genes were previously reported as upregulated during late induction (Bélanger et al., 2018), providing cross-study validation of the observed patterns. Among *PRX*s, only one gene (HORVU.MOREX.r2.3HG0231680.1; a B-type/1-Cys peroxiredoxin orthologue of Arabidopsis) showed a clear association with efficient ME, being strongly induced at stage III in Igri and reaching high abundance (>200 FPKM). Plant 1-Cys *PRXs*, though less characterized, interact with TRX/GRX systems to mitigate ROS, transduce stress signals, and modulate metabolism, under severe stress their peroxidase activity can switch to a chaperone function (Dietz et al., 2006; Kim et al., 2011; Liebthal et al., 2018). Its strong induction in the responsive cultivar points to a role in proteostasis during rapid proliferative remodeling.

In addition to ME-associated induction, a substantial subset of *TRX*/*GRX*/*PRX* genes was strongly expressed in mature pollen but showed little or no responsiveness during ME induction, indicating that thiol–redox regulation also supports late gametophytic development independently of embryogenic reprogramming. This separation of expression patterns suggests functional specialization within thiol–redox families, with distinct members contributing either to pollen maturation and stress tolerance or to the redox remodeling required for embryogenic transition.

### 4.5 Confirmation of the important role of GST in ME induction

GSTs likely contribute to ME through multiple mechanisms. They catalyzing S-glutathionylation—a reversible modification that shields protein thiols from irreversible oxidation while modulating protein function (Sevilla et al., 2023). GSTs detoxify stress-derived metabolites and participate in biosynthetic pathways (Piślewska-Bednarek et al., 2018). Although some plant GSTs show limited glutathionylating activity (Micic et al., 2024), the family’s size underscores functional breadth. In the present study, *GST* transcription was progressively activated during ME induction, with a pronounced enrichment at stage III in the responsive cultivar Igri. This pattern closely parallels observations made in the independently characterised ME-responsive cultivar Gobernadora (Bélanger et al., 2018), in which late-stage *GST*s induction was likewise associated with successful embryogenic development. The concordant late *GST* activation in both Igri and Gobernadora therefore confirms their shared responsive phenotype and provides cross-study validation that stage-III GST upregulation is a hallmark of effective embryogenic reprogramming.

Early studies identified *GST*s among the first ME-responsive genes in barley (Vrinten et al., 1999), and subsequent studies documented dynamic *GST* expression across induction and its association with plant regeneration capacity (Maraschin et al., 2006; Muñoz-Amatriaín et al., 2006, Muñoz-Amatriaín et al., 2009; Joosen et al., 2007; Malik et al., 2007; Tsuwamoto et al., 2007; Jacquard et al., 2009; Sánchez-Díaz et al., 2013; Żur et al., 2014b; Bélanger et al., 2020). Our findings extend these observations by resolving stage- and cultivar-specific programs and implicating elevated GST activity as a hallmark of the responsive trajectory.

### 4.6 A model for redox control during ME

**Figure 6:**
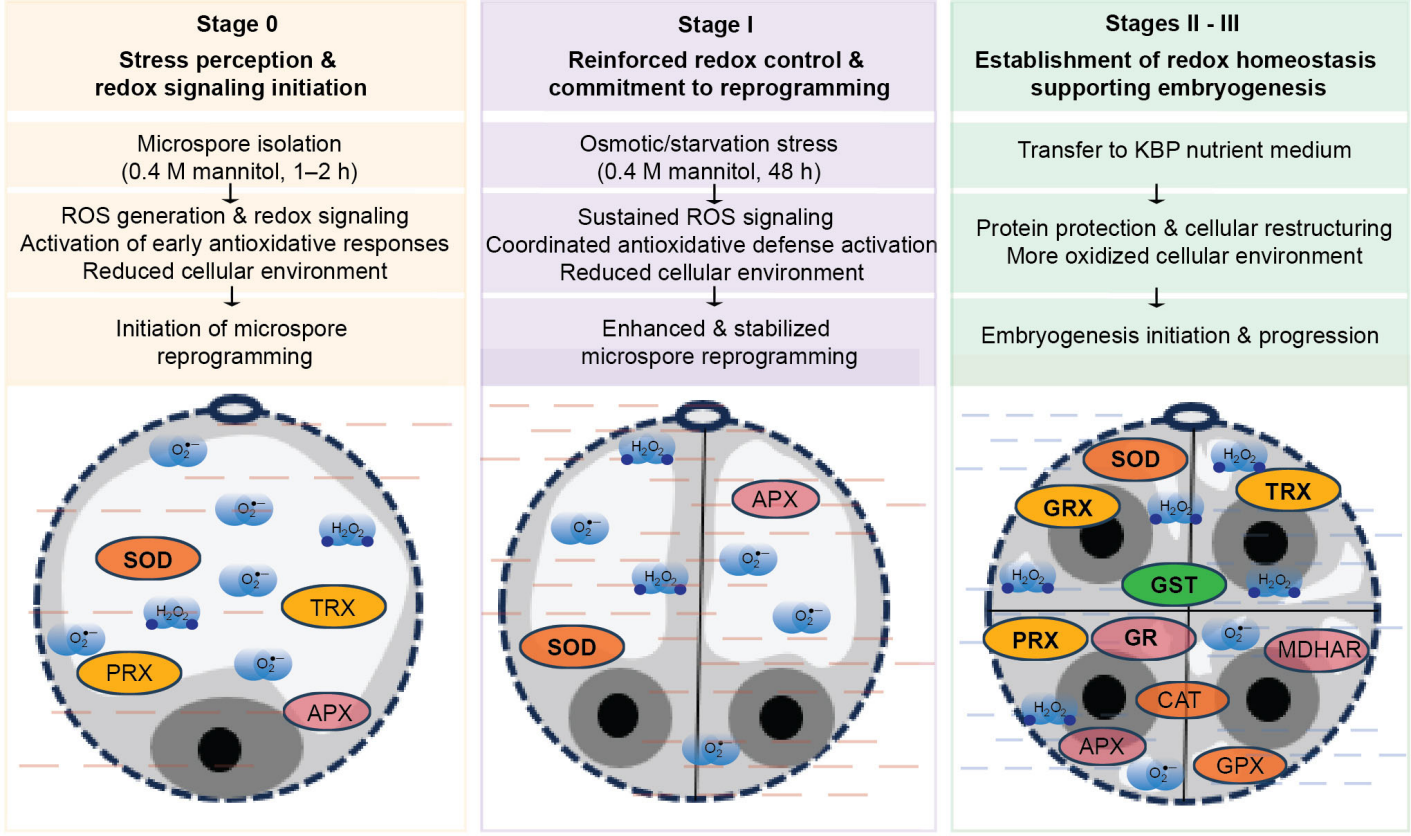
Conceptual model summarizing transcriptome-derived redox regulatory dynamics underlying barley microspore embryogenesis induction. Short-term osmotic stress (1–2 h of 0.4 M mannitol) applied during anthers collection together with the stress induced by mechanical isolation of microspores (Stage 0) triggers reactive oxygen species (ROS) production and redox signaling, leading to activation of core antioxidant defenses and initiation of microspore reprogramming. This early phase is characterized by elevated expression of *SUPEROXIDE DISMUTASES* (*SOD*s) and *ASCORBATE PEROXIDASES* (*APX*s), together with induction of thiol-based redox regulators, including *THIOREDOXINS* (*TRX*s) and *PEROXIREDOXINS* (*PRX*s). Prolonged osmotic/starvation stress (Stage I) sustains ROS signaling and reinforces coordinated antioxidant responses, supporting commitment to and stabilization of the reprogramming process. Following transfer to nutrient medium (Stages II–III), a broader redox network becomes established, incorporating *CATALASES* (*CAT*s), *GLUTATHIONE PEROXIDASES* (*GPX*s), *MONODEHYDROASCORBATE REDUCTASES* (*MDHAR*s), *GLUTATHIONE REDUCTASES* (*GR*s), *GLUTAREDOXINS* (*GRX*s), *TRX*s, *PRX*s, and *GLUTATHIONE S-TRANSFERASES* (*GST*s). This expanded network supports protein protection, redox homeostasis, cellular restructuring, and initiation and progression of embryogenesis. These redox regulatory processes represent a core transcriptional framework shared by both cultivars (Igri and Golden Promise) and are essential for microspore survival and developmental reprogramming. Comparative transcriptomic analysis further reveals cultivar-specific quantitative differences: the responsive cultivar Igri displays higher cumulative transcript abundance of multiple antioxidant and detoxification gene families, including stronger late-stage induction of *GR*s, enhanced activation of thiol-based redox components (*TRX*s/*GRX*s/*PRX*s), and a more pronounced *GST*-mediated detoxification response (highlighted in bold). Intensive utilization of GSH may shift the cellular redox balance toward a more oxidized state conducive to continued embryogenic development likely underpins successful embryogenesis in Igri. Enzyme family names denote transcriptional regulation inferred from RNA-seq analyses and do not represent direct measurements of enzymatic activity.

Integrating transcript abundance patterns across antioxidant and redox gene families with their functional relationships (**Figure 6**), we propose a staged model of redox control during ME. An early phase, common to both cultivars, is characterized by activation of core antioxidant defenses that buffer the oxidative burst associated with microspore isolation and osmotic/starvation stress, thereby enabling cell survival and entry into developmental reprogramming. This phase is marked by stable or moderately elevated expression of *SOD*s, *APX*s, and selected *CAT* and *GPX* isoforms, consistent with maintenance of basal ROS detoxification and redox homeostasis.

In contrast, the embryogenically responsive trajectory represented by Igri shows a second, coordinated redox reinforcement at later stages (II–III), coincident with the initiation of the embryogenic developmental program. This late phase involves enhanced glutathione recycling via GR, pronounced activation of GSTs, and selective deployment of thiol–redox regulators (TRXs, GRXs, and PRXs), rather than a global increase in antioxidant capacity (highlighted in **Figure 6**). Such targeted engagement of thiol-based redox hubs is well suited to fine-tune protein redox status, preserve proteostasis, and sustain redox-sensitive signaling pathways during rapid cell proliferation and multicellular structure formation. We propose that this program maintains a signaling-competent O_2_^•–^ and H_2_O_2_ pools while preventing oxidative damage, thereby favoring cell-cycle progression and ELS development. Intensive utilization of GSH as an electron donor by GST, GRX, and GPX enzymes may shift the cellular redox balance toward a more oxidized state conducive to continued embryogenic development (Stasolla, 2010). By contrast, the recalcitrant cultivar Golden Promise may over-scavenge or mistime key steps, attenuating ROS signaling required for the fate switch.

The presented model appears to be supported by multiple experimental data (as referenced above); however, further validation could be achieved via (i) targeted perturbation of candidate nodes (e.g. CAT/GPX inhibition or GSH supplementation), (ii) real-time compartment-specific redox imaging (e.g. roGFP-based reporters), and (iii) genetic manipulation of discriminative *TRX*/*GRX*/*PRX* and *GST* candidates.

## 5 Conclusions

Our *in silico* analysis shows that transcription of antioxidant and redox genes is dynamically remodeled during ME induction in barley, underscoring the centrality of redox homeostasis in successful microspore reprogramming. We delineate, for the first time, the breadth and stage-resolved coordination of this regulatory network in response to ME-associated stress and highlight candidate contributors—including *MDHAR*, *GR*, specific *TRX*/*GRX* and *PRX* members, and multiple *GSTs* (**Figure 6**). Contrasting responsiveness between cultivars enabled the nomination of candidate molecular markers potentially linked to embryogenic competence. These findings provide mechanistic insight into ME and offer practical leads for improving DH production in cereals.

## Data Availability

The datasets presented in this study can be found in online repositories. The names of the repository/repositories and accession number(s) can be found in the article/**Supplementary Material**.
