# Supplementary material for: Transcriptomic identification of potential antioxidative enzyme regulators of the gametophytic-to-embryogenic switch in barley microspores
Source: Front Plant Sci. 2026 Jan 21;16:1735720. doi: 10.3389/fpls.2025.1735720 (PMC12868223; doi:10.3389/fpls.2025.1735720)
Supplement: Supplementary file 1 [file SupplementaryFile1.pdf]

Supplementary Information

Transcriptomic identification of potential antioxidative enzyme regulators of the gametophytic-to-embryogenic switch in barley microspores

Anna Nowicka[1,2], Zbyněk Milec[3], Monika Krzewska[1], Przemysław Kopeć[1], Agnieszka Springer[1], Ewa Dubas[1], Iwona Żur[1]*

[1]The Franciszek Go´ rski Institute of Plant Physiology Polish Academy of Sciences, Kraków, Poland

[2]Centre of Plant Structural and Functional Genomics, Institute of Experimental Botany (IEB), Czech Academy of Sciences (CAS), Olomouc, Czechia

[3]Laboratory of Ecological Plant Physiology, Global Change Research Institute, Czech Academy of Sciences (CAS), Brno, Czechia

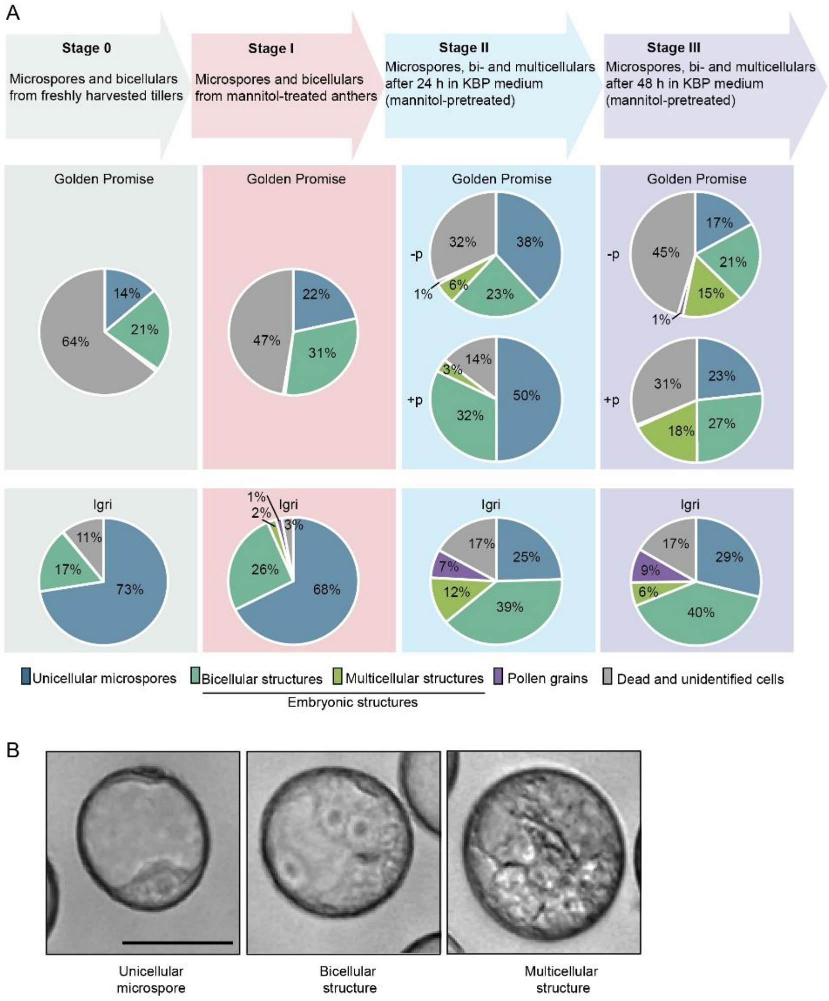

Figure S1. Phenotypic progression of barley microspores during induction of microspore embryogenesis (ME). **This figure complements Fig. 1.**

(A) Composition of cell types at four stages of the induction protocol in two barley cultivars—Golden Promise (recalcitrant) and Igri (responsive). Stages: 0, microspores isolated from freshly harvested spikes/anthers; I, microspores isolated from anthers pre-treated with 0.4 M mannitol for 48 h at 21 °C; II, structures isolated from mannitol-pre-treated anthers and cultured for 24 h in KBP medium; III, structures isolated from mannitol-pre-treated anthers and cultured for 48 h in KBP medium. Pie charts show the proportions of uninucleate microspores; bicellular structures after symmetric division; multicellular structures arising from continued symmetric divisions; pollen grains with two unequal nuclei following asymmetric division; and dead/unidentified cells (colour key at bottom). For Golden Promise, stages II–III are shown for cultures without (−p) and with (+p) co-cultivation with wheat pistils; RNA-seq for stages II–III was performed on +p samples. Quantitative data redrawn from Nowicka et al. (2024).

(B) Representative micrographs of a uninucleate microspore, a bicellular structure and a multicellular structure during ME induction. Scale bar = 20 µm.

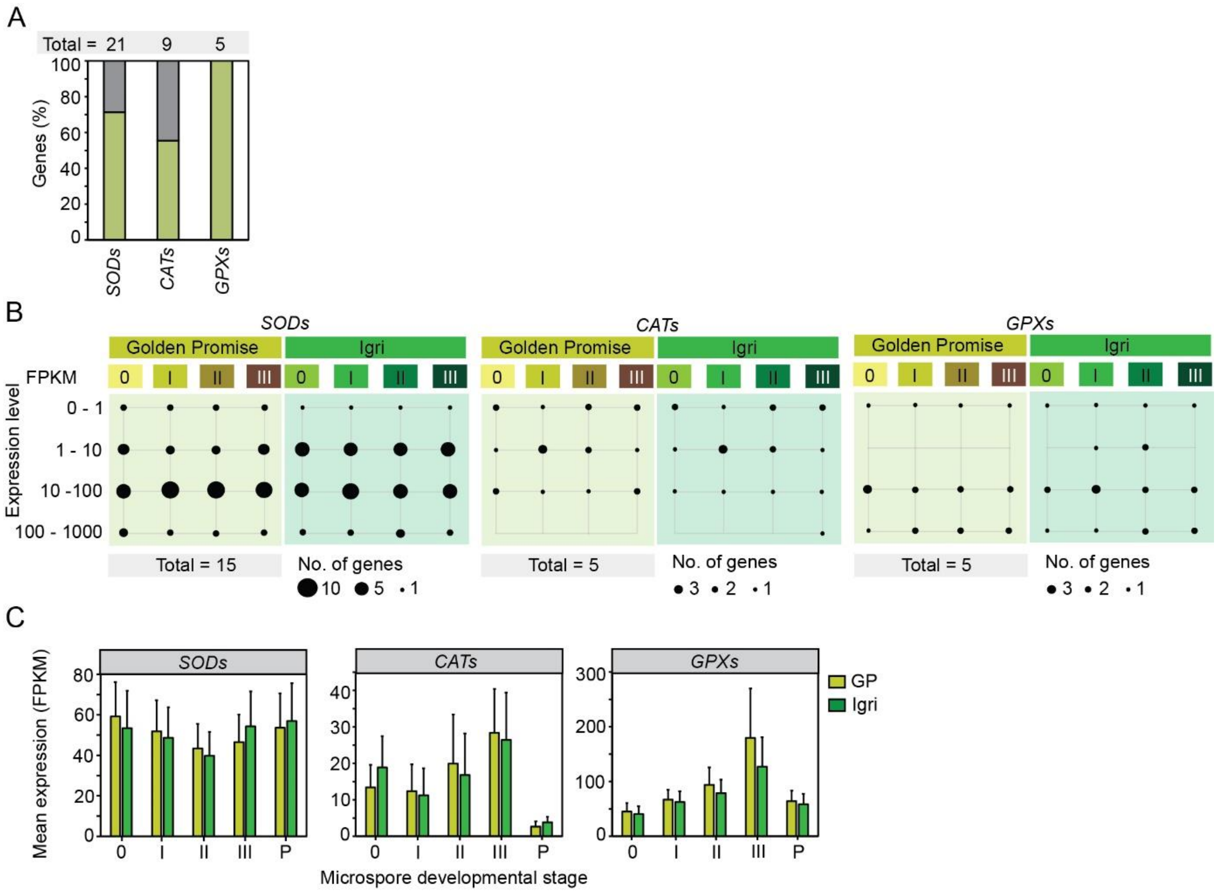

Figure S2. Expression profiles of core antioxidant enzyme gene families during microspore embryogenesis (ME) in Golden Promise and Igri.

Core antioxidant enzymes analysed include *SUPEROXIDE DISMUTASE*s (*SOD*s), *CATALASE*s (*CAT*s) and *GLUTATHIONE PEROXIDASE*s (*GPX*s). **This figure complements Fig. 2.**

(A) Fraction of annotated genes in each family that show detectable expression (*FPKM* > 0) versus no detectable expression (FPKM = 0) in at least one sampled stage. Numbers above bars indicate the total number of annotated genes per family.

(B) Distribution of family members across transcript abundance classes at each stage in Golden Promise and Igri. Genes were grouped by FPKM into very low (0–1), low (1–10), moderate (10–100) and high (100–1,000) expression classes. Circle size reflects the number of genes within each expression class and stage.

(C) Mean transcript abundance (FPKM; mean ± SE) for each gene family in Golden Promise (GP) and Igri. Only genes with detectable expression (FPKM > 0) in at least one stage in either cultivar were included. Stages 0–III correspond to successive steps of ME induction, whereas P denotes bicellular pollen representing the gametophytic pathway.

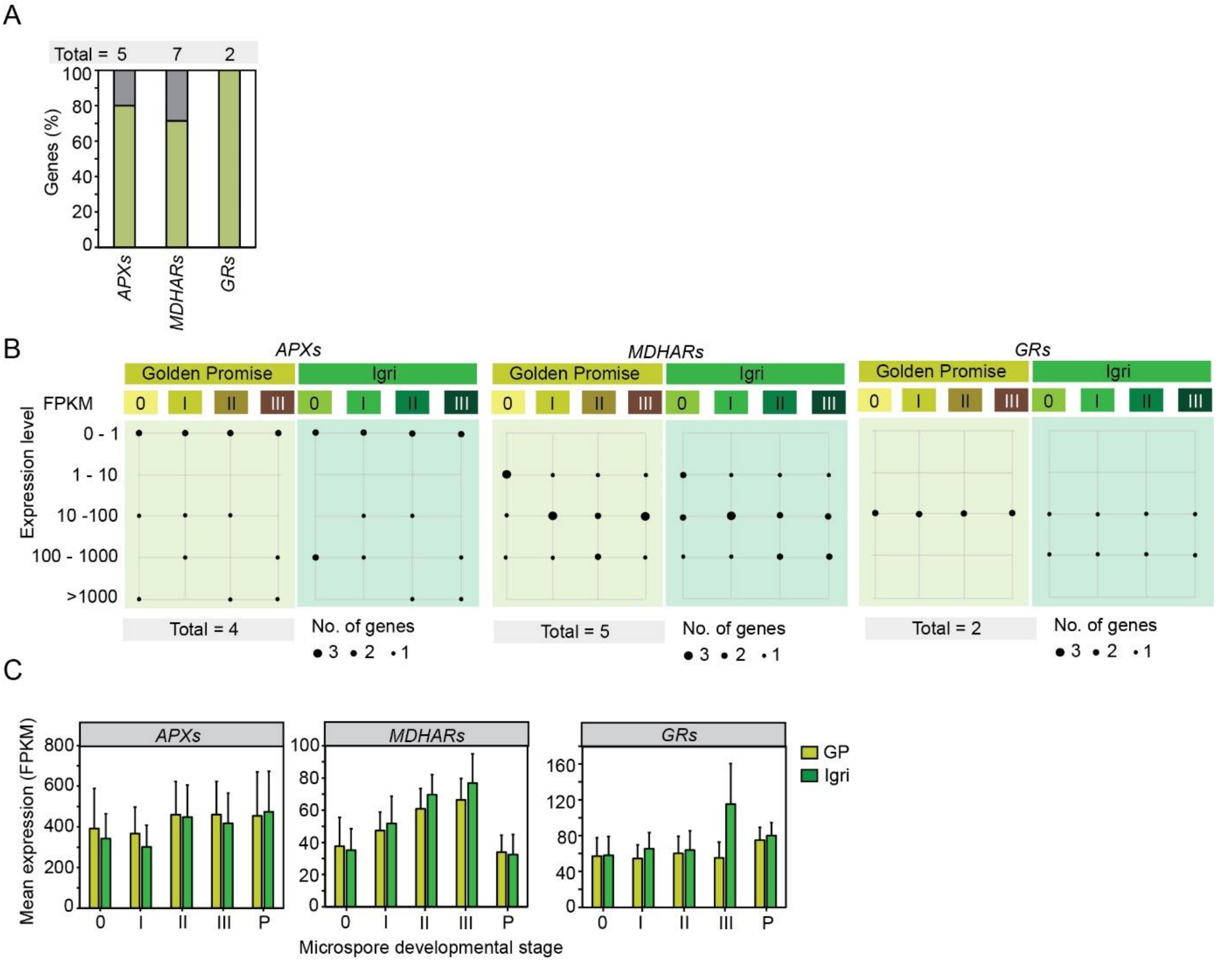

Figure. S3. Expression profiles of ascorbate–glutathione (ASC–GSH) cycle gene families during microspore embryogenesis (ME) in Golden Promise and Igri.

Families include *ASCORBATE PEROXIDASE*s (*APX*s), *MONODEHYDROASCORBATE REDUCTASE*s (*MDHAR*s), and *GLUTATHIONE REDUCTASE*s (*GR*s). **This figure complements Fig. 3.**

(A) Fraction of annotated genes per family with detectable expression (FPKM > 0) versus no detectable expression (FPKM = 0) in at least one sampled stage. Numbers above bars indicate the total number of annotated genes in each family.

(B) Stage-wise distribution of family members across transcript abundance classes in Golden Promise and Igri. Genes were grouped by FPKM into very low (0–1), low (1–10), moderate (10–100), high (100–1,000), and very high (>1,000) expression classes. Circle size is proportional to the number of genes in each class at each stage.

(C) Mean transcript abundance (FPKM; mean ± SE) for each gene family in Golden Promise (GP) and Igri. Only genes with detectable expression (FPKM > 0) in at least one stage in either cultivar were included. Stages 0–III represent successive steps of ME induction, whereas P denotes bicellular pollen (gametophytic development).

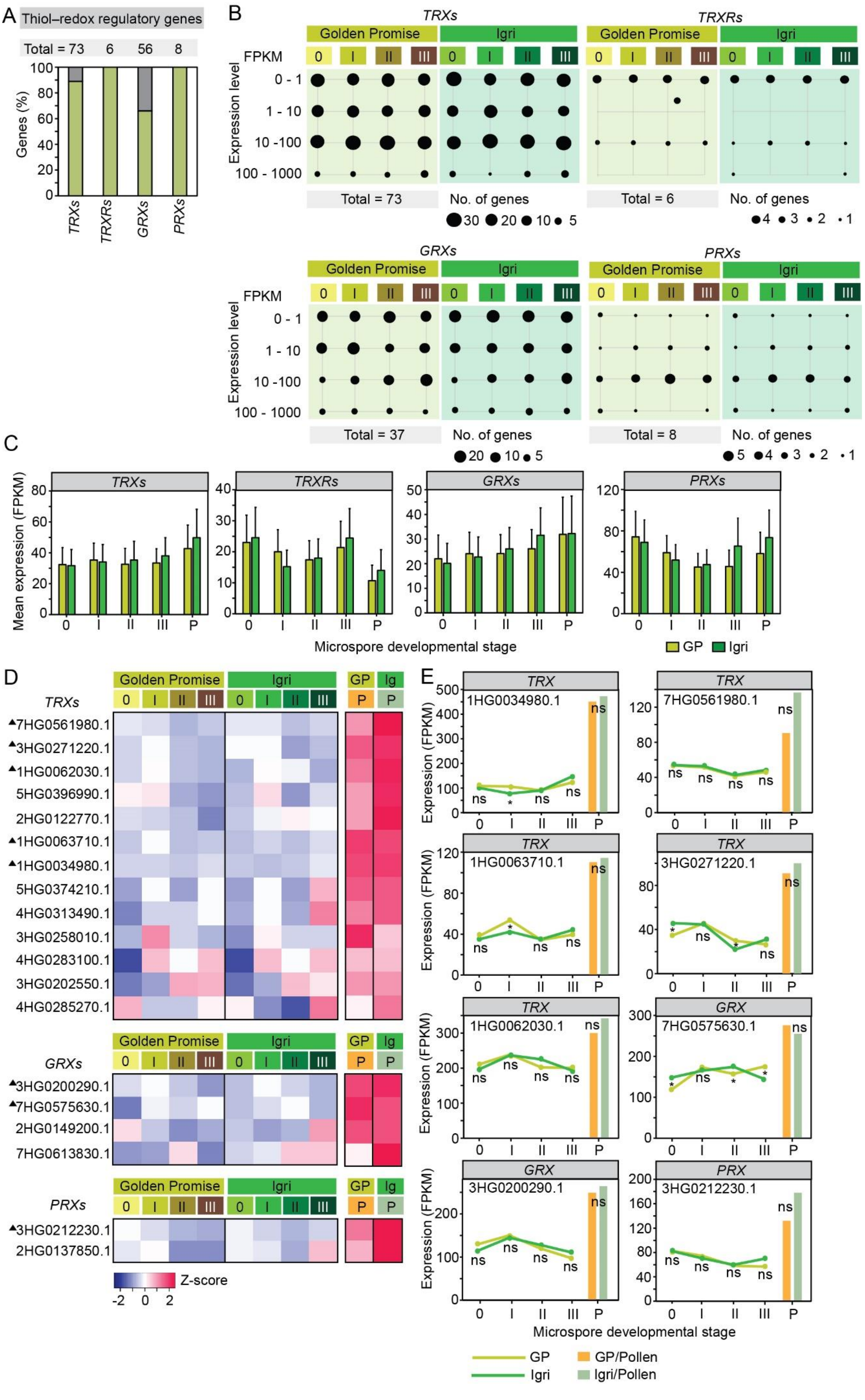
A
Thiol–redox regulatory genes
Total = 73
6
56
8
Genes (%)
TRXs
TRXRs
GRXs
PRXs
B
TRXs
TRXRs
GRXs
PRXs
Golden Promise
Igri
FPKM
0
I
II
III
Expression level
0 - 1
1 - 10
10 -100
100 - 1000
Total = 73
No. of genes
30
20
10
5
Total = 6
4
3
2
1
Total = 37
Total = 8
C
Mean expression (FPKM)
P
Microspore developmental stage
GP
Igri
D
Z-score
-2
0
2
Ig
7HG0561980.1
3HG0271220.1
1HG0062030.1
5HG0396990.1
2HG0122770.1
1HG0063710.1
1HG0034980.1
5HG0374210.1
4HG0313490.1
3HG0258010.1
4HG0283100.1
3HG0202550.1
4HG0285270.1
3HG0200290.1
7HG0575630.1
2HG0149200.1
7HG0613830.1
3HG0212230.1
2HG0137850.1
E
TRX
GRX
PRX
Expression (FPKM)
ns
*
GP/Pollen
Igri/Pollen

Figure S4. Expression profiles of thiol–redox regulatory genes during microspore embryogenesis (ME) and gametophytic pollen development in barley.

Thiol–redox regulatory gene families analysed include *THIOREDOXIN*s (*TRX*s), *THIOREDOXIN REDUCTASE* (*TRXR*s*),* *GLUTAREDOXIN*s (*GRX*s) and *PEROXIREDOXIN*s (*PRX*s*).* **This figure complements Fig. 4.**

(A) Proportion of annotated genes per family showing detectable expression (FPKM > 0) versus no detectable expression (FPKM = 0) in at least one sampled stage. Numbers above bars indicate the total number of annotated genes per family.

(B) Stage-wise distribution of family members across transcript abundance classes in Golden Promise and Igri. Genes were binned by FPKM into very low (0–1), low (1–10), moderate (10–100) and high (100–1,000) expression classes. Circle size is proportional to the number of genes in each class at each stage.

(C) Mean transcript abundance (FPKM; mean ± SE) for each gene family in Golden Promise (GP) and Igri. Only genes with detectable expression (FPKM > 0) in at least one stage in either cultivar were included. Stages 0–III represent successive steps of ME induction, whereas P denotes bicellular pollen (gametophytic development).

(D) Heatmaps of selected pollen-enriched thiol–redox genes (13 *TRX*s, 4 *GRX*s and 2 *PRX*s; HORVU.MOREX.r2 identifiers) showing high expression in bicellular pollen (P) and low expression across ME stages. Values are shown as row z-scores. Black triangles mark genes highlighted in panel E and discussed in the main text.

(E) Expression trajectories for selected *TRX*, *GRX* and *PRX* genes across ME stages (line plots) and in bicellular pollen (P; bar plots). Asterisks indicate significant differential expression between cultivars at a given stage (DESeq2; FDR-adjusted $P < 0.05$); ns indicates non-significance.

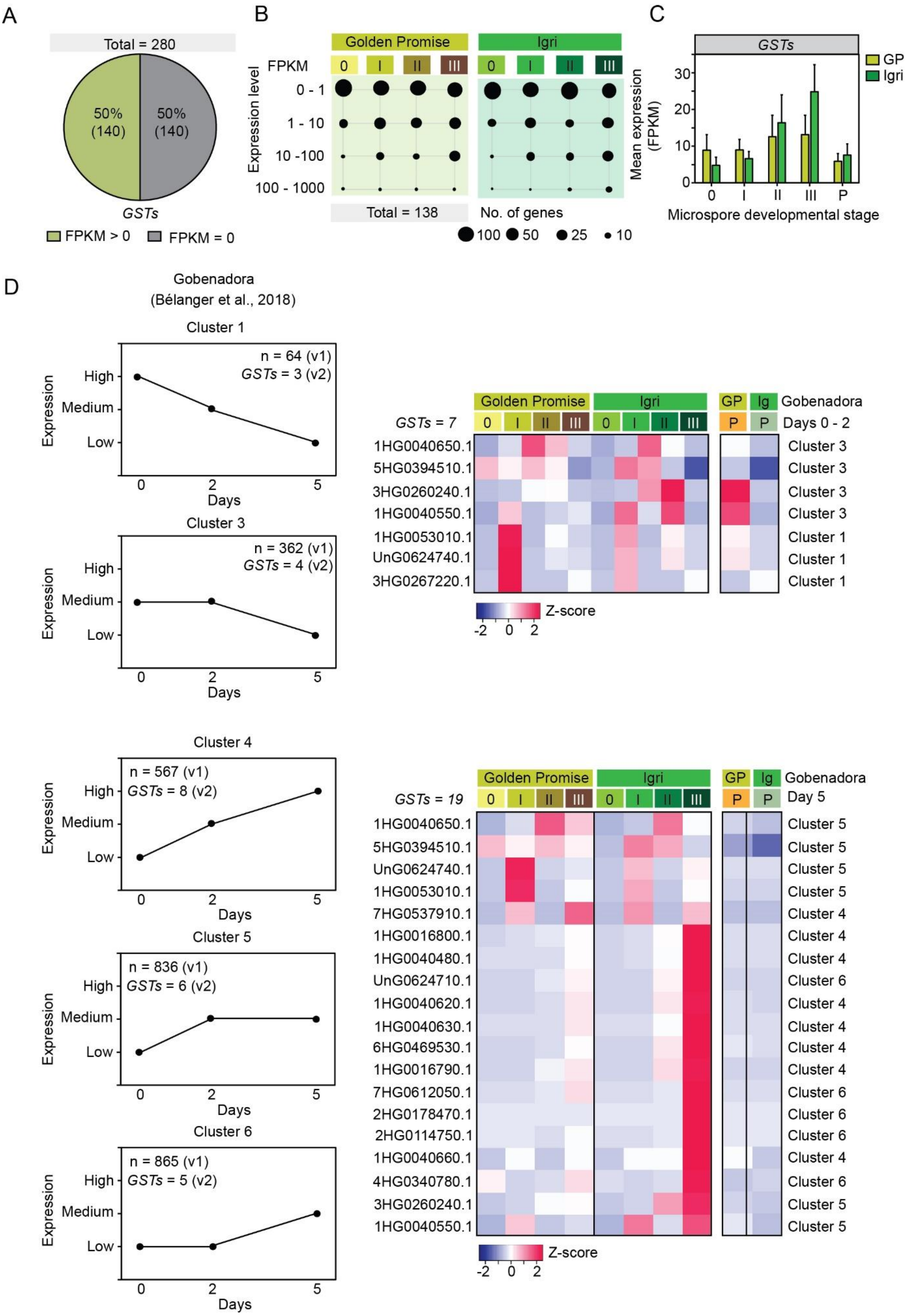

A
Total = 280
50% (140)
50% (140)
GSTs
FPKM > 0
FPKM = 0
B
Golden Promise
Igri
FPKM
0
I
II
III
Expression level
0 - 1
1 - 10
10 -100
100 - 1000
Total = 138
No. of genes
100
50
25
10
C
GSTs
GP
Igri
Mean expression (FPKM)
Microspore developmental stage
P
D
Gobenadora
(Bélanger et al., 2018)
Cluster 1
n = 64 (v1)
GSTs = 3 (v2)
Expression
High
Medium
Low
Days
Cluster 3
n = 362 (v1)
GSTs = 4 (v2)
GSTs = 7
Gobenadora
Days 0 - 2
GP
Ig
1HG0040650.1
5HG0394510.1
3HG0260240.1
1HG0040550.1
1HG0053010.1
UnG0624740.1
3HG0267220.1
Cluster 3
Cluster 1
Z-score
Cluster 4
n = 567 (v1)
GSTs = 8 (v2)
Cluster 5
n = 836 (v1)
GSTs = 6 (v2)
Cluster 6
n = 865 (v1)
GSTs = 5 (v2)
GSTs = 19
Day 5
1HG0040650.1
5HG0394510.1
UnG0624740.1
1HG0053010.1
7HG0537910.1
1HG0016800.1
1HG0040480.1
UnG0624710.1
1HG0040620.1
1HG0040630.1
6HG0469530.1
1HG0016790.1
7HG0612050.1
2HG0178470.1
2HG0114750.1
1HG0040660.1
4HG0340780.1
3HG0260240.1
1HG0040550.1
Cluster 5
Cluster 4
Cluster 6

Figure S5. Expression profiles of *GLUTATHIONE S-TRANSFERASE* (*GST*) genes during microspore embryogenesis (ME) in barley. **This figure complements Fig. 5.**

(A) Proportion of annotated *GST* genes with detectable expression (FPKM > 0) versus no detectable expression (FPKM = 0) in at least one sampled stage.

(B) Stage-wise distribution of GST genes across transcript abundance classes in Golden Promise and Igri. Genes were binned by FPKM into very low (0–1), low (1–10), moderate (10–100), and high (100–1,000) expression classes. Circle size is proportional to the number of genes in each class at each stage.

(C) Mean transcript abundance (FPKM; mean ± SE) of expressed *GST* genes in Golden Promise (GP) and Igri. Only genes with detectable expression (FPKM > 0) in at least one stage in either cultivar were included. Stages 0–III represent successive steps of ME induction, whereas P denotes bicellular pollen (gametophytic development).

(D) Cross-study comparison with the responsive cultivar Gobernadora (Bélanger et al., 2018). Left panels show the expression trajectories of Gobernadora gene clusters across 0, 2 and 5 days in culture, indicating the total number of genes per cluster (n) and the number of *GST* genes assigned to each cluster. Right panels show heatmaps of *GST*s identified in Gobernadora and their corresponding expression patterns in Golden Promise and Igri (HORVU.MOREX.r2 identifiers), including expression in bicellular pollen (P). Expression values are shown as row z-scores. Barley reference annotation versions are indicated as v1 (Mascher et al., 2017) and v2 (Monat et al., 2019).
